# Effects of a Text Messaging Smoking Cessation Intervention Among Online Help Seekers and Primary Health Care Visitors in Sweden: Protocol for a Randomized Controlled Trial Using a Bayesian Group Sequential Design

**DOI:** 10.2196/23677

**Published:** 2020-12-03

**Authors:** Marcus Bendtsen, Kristin Thomas, Catharina Linderoth, Preben Bendtsen

**Affiliations:** 1 Department of Health, Medicine and Caring Sciences Linköping University Linköping Sweden; 2 Department of Medical Specialist Motala Sweden

**Keywords:** smoking cessation, text messaging, online help seekers, primary healthcare units, randomized controlled trial, Bayesian group sequential design, Bayesian, smoking, protocol, intervention

## Abstract

**Background:**

A steady decline of the smoking prevalence in Sweden has been recorded over the past decade; however, people still start and continue to smoke. There is a need for effective smoking cessation interventions that can scale to a national level and that are designed to reach individuals requiring smoking cessation support in the general population.

**Objective:**

Previous randomized controlled trials of smoking cessation interventions among high school and university students in Sweden have found consistent evidence that text messaging interventions are effective in helping students quit smoking. However, there are no studies that investigate the effects of text messaging interventions in a more general population. The objective of this study is to estimate the effects of a text messaging intervention on individuals seeking help to quit online and individuals visiting primary health care units.

**Methods:**

A 2-arm, parallel-group (1:1), randomized controlled trial will be employed to address the study objectives. The trial will follow a Bayesian group sequential design. Recruitment will be conducted using online advertisement (Google, Bing, and Facebook) and through health care professionals at primary health care units. All participants will receive treatment as usual; however, participants who are allocated to the intervention arm will also be given access to a 12-week text message smoking cessation intervention. Primary outcomes are 8-week prolonged abstinence and 4-week point prevalence, measured 3 months and 6 months postrandomization. Mediator variables (self-efficacy, importance, and know-how) will be measured to estimate causal mediation models.

**Results:**

Recruitment commenced in September 2020 and will not exceed 24 months. This means that a complete dataset will be available at the latest towards the end of 2022. We expect to publish the findings from this trial by June 2023.

**Conclusions:**

This trial will further our understanding of the effects of text messaging interventions among a more general population than has previously been studied. We also aim to learn about differential effects between those who seek support online and those who are given facilitated support at primary health care units. Trial recruitment is limited to the Swedish population; however, a strength of this study is the pragmatic way in which participants are recruited. Through online advertisements, individuals are recruited in reaction to their own interest in seeking help to quit. At primary health care units, individuals who were not necessarily looking for smoking cessation support are given information about the trial. This closely mimics the way the intervention would be disseminated in a real-world setting and may therefore strengthen the argument of generalizability of findings.

**Trial Registration:**

ISRCTN 13455271; http://www.isrctn.com/ISRCTN13455271.

**International Registered Report Identifier (IRRID):**

PRR1-10.2196/23677

## Introduction

### Background and Rationale

In 2017, the Global Burden of Diseases, Injuries, and Risk Factors Study found that globally, the second leading risk factor for disability adjusted life years was smoking [1], closely following high systolic blood pressure among the factors considered. Smokers are at higher risk of contracting several noncommunicable diseases, including cancer, diabetes, and cardiovascular and respiratory diseases. Despite strong evidence for the negative consequences of smoking, it continues to be a legal substance that harms and kills many individuals when used as intended by manufacturers [2].

A steady decline of the smoking prevalence in Sweden has been recorded over the past decade [3]. The most recent data from 2018 indicate that the prevalence rate was as low as 7% in the general population, lower than the 12% prevalence rate of daily use of snus (a type of tobacco placed under the lip), but higher than vaping, which is only used by 0.6% of Swedes on a daily basis [4]. This means that we are closer than ever to eradicating one of the most important causes of disease in Sweden. However, as there still are smokers and young individuals still start smoking, there is a need for effective smoking cessation interventions that can scale to a national level and that are designed to reach individuals requiring smoking cessation support in the general population.

Mobile phone–based interventions such as text messaging interventions could potentially have far reach among those who may benefit, in particular due to their reliance on standard technology and the high mobile phone ownership in Sweden. Typically, these interventions consist of a series of messages sent to participants’ mobile phones over the course of 8-12 weeks. The messages motivate participants to make a quit attempt and then reinforce and support this decision throughout the intervention period. In addition, text messaging interventions may also increase access to education and support services that promote smoking cessation [5].

Several randomized controlled trials (RCTs) have been conducted to estimate the effects of text messaging interventions for smoking cessation [5-11], notably the txt2stop trial [9] (n=5800), which found strong evidence in favor of the intervention with respect to both biochemically verified abstinence (odds ratio [OR] 2.20, 95% CI 1.80-2.68, *P*<.001) and self-reported abstinence (OR 1.47, 95% CI 1.40-1.66, *P*<.001). Three meta-analyses concluded that text messaging interventions have a positive effect on smoking cessation: One reported a summary effect size (Hedges’ g) of 0.25 (95% CI 0.13-0.38) [6], the second meta-analysis reported an overall summary OR of 1.37 (95% CI 1.25-1.51) of smoking cessation in favor of text messaging interventions [5], and the third analysis similarly found that quit rates were higher among those who had access to text messaging interventions (OR 1.36, 95% CI 1.23-1.51) [7]. Thus, there exists a relatively strong body of evidence for the average treatment effect of text messaging interventions.

In Sweden, our research group has previously conducted RCTs of smoking cessation interventions among high school [12,13] and university students [11,14-16]. Here, we found consistent evidence that a text message intervention was effective in increasing the prevalence of smoking abstinence. We are also currently conducting an RCT of a text message smoking cessation intervention tailored to patients undergoing elective surgery [17,18]. However, these interventions have recruited participants from well-defined contexts (ie, high schools, university campuses, and surgical departments) but have not taken a broader approach to recruitment in the general population. Also, there have not been any studies in Sweden of text message smoking cessation interventions targeting the general population.

### Objectives

The objective of this study is to estimate the effects of a text message smoking cessation intervention as a complement to treatment as usual in the general population of Sweden. In addition, the study aims to gain knowledge on the differences between individuals recruited from 2 distinct settings: online advertisement and primary health care units. In particular, the objectives of the trial are to (1) estimate the effects of a text messaging smoking cessation intervention on prevalence rates of smoking abstinence compared to individuals without access to the intervention; (2) estimate the degree to which the total effect is mediated through motivation, importance, and know-how; (3) estimate the degree to which the total effect is moderated by the mode of recruitment: online advertisements versus primary health care units; and (4) investigate differences in baseline characteristics between participants recruited through online advertisements and participants recruited through primary health care units.

### Trial Design

A 2-arm, parallel-group (1:1) RCT will be employed to address the study objectives. The trial will follow a Bayesian group sequential design [19-21] (see Sample Size). All participants will receive treatment as usual; however, participants who are allocated to the intervention arm will also be given access to a text message smoking cessation intervention.

## Methods

This trial was preregistered on July 27, 2020 (ISRCTN13455271) and received ethical approval from the Swedish Ethical Review Authority (Dnr 2020–01427, 2020-06-16).

### Participants, Interventions, Outcomes

#### Study Setting and Recruitment

Recruitment will take place in 2 distinct settings. First, online advertisements on Google, Bing, and Facebook (restricted to Sweden) will be used to recruit individuals who are seeking help to quit smoking. Individuals clicking on the advert will be taken to the study website where information will be presented about the study and how to sign up. Second, health care professionals at participating primary health care units across Sweden will advertise the trial to patients through printed media (eg, flyers, leaflets, business cards, posters). The printed media will contain information about the study and how to sign up.

Regardless of setting, individuals will sign up for the trial by sending a text message to a dedicated telephone number. Within 5 minutes, they will receive a text message in response, with a hyperlink to an informed consent form (see Multimedia Appendix 1). Participants consenting to take part in the trial will immediately be redirected to the baseline questionnaire, after which eligible participants will be randomized (see Assignment of Interventions).

#### Eligibility Criteria

Individuals self-reporting smoking at least 1 cigarette per week and who are aged ≥18 years will be eligible for the trial. Individuals who self-report not smoking or doing so less than weekly or are aged <18 years will be explicitly excluded from the trial. The majority of study information and all questionnaires will be delivered to participants through a mobile phone and will be in Swedish; thus, participants without access to a mobile phone and who do not comprehend Swedish well enough to sign up for the trial will be implicitly excluded.

#### Interventions

Both the intervention and control groups will be given treatment as usual, and neither will be restricted from using other available smoking cessation aids. The intervention group will in addition be given access to a text message intervention.

Treatment as usual will in this trial be defined as follows. For participants recruited through online advertisements, it is defined as referral to national quit lines (sluta-röka-linjen [22]) and general information about smoking and health (1177 Vårdguiden [23]). For participants recruited through primary health care units, it is defined as referral to national quit lines (sluta-röka-linjen [22]) and referral to general information about smoking and health (1177 Vårdguiden [23]). In addition, primary health care units will offer all smokers a meeting with a nurse or smoking cessation specialist to have a conversation about smoking cessation and health.

Participants allocated to the intervention group will be given access to a text message intervention. Two versions of the intervention exist: one general version and one that has been tailored specifically for individuals undergoing elective surgery. Both versions are based on findings from our previous research [11-18]. The elective surgery intervention will be allocated to participants in the intervention group who report having elective surgery planned in the next 3 months.

Both versions of the intervention consist of a 12-week text message program with messages sent to participants’ mobile phones on a daily basis. Over the first few weeks, all participants will receive 2-4 messages per day, which will be reduced to 2 messages per day during the middle part of the intervention and further reduced to 1 message per day during the latter part of the intervention. The content of the messages is primarily informational and encouraging, and some messages ask participants to do certain tasks, such as throw away ashtrays. None of the messages ask participants to respond, but participants can request extra supportive messages by sending a text message with 1 of 3 keywords: weight, relapse, or craving. A message is then sent back to participants with specific information about potential weight gain, what to do if one relapses and has a cigarette, or help if they are experiencing nicotine cravings.

Unique for the elective surgery version is that some of the messages include hyperlinks that take the participants to interactive web-based modules. There is a total of 9 such modules and, throughout the intervention period, participants will be reminded to revisit previously completed modules.

Briefly, the 9 interactive modules included in the elective surgery version of the intervention are (1) set of tips and tasks, of which participants choose 5 that suit them; (2) set of reasons to quit smoking, of which the participants choose 5 (or enter their own reason); (3) series of questions that result in a plan for how to deal with certain situations in which the temptation to smoke is increased, such as “When I have had my dinner, I will have a piece of fruit” or “While I am waiting for the bus, I will listen to music.”; (4) set of information boxes that relate to withdrawal symptoms; (5) set of tips that participants choose, or enter on their own, on what to do when craving cigarettes; (6) set of information boxes with information about what happens to the human body after smoking cessation; (7) set of information boxes with information about good habits that can replace the smoking habit; (8) series of questions that lead to suggestions of physical activities that the participant might want to try in order to improve his or her health further and relieve abstinence; and (9) pros and cons list created by the participant.

#### Outcomes

All outcomes will be self-reported through questionnaires. Please see Multimedia Appendix 2 for all questionnaires used in the trial.

The primary outcomes include prolonged abstinence, following the Russell standard definition of not having smoked more than 5 cigarettes in the past 8 weeks (thus allowing for a 4-week grace period) [24]. The abstinence period will be adjusted to 5 months at the 6-month follow-up. Another primary outcome is the point prevalence of smoking abstinence, defined as not smoking any cigarette during the past 4 weeks, as recommended by the Society for Research on Nicotine and Tobacco [25].

Secondary outcomes include the 7-day point prevalence of complete smoking abstinence, number of cigarettes smoked weekly (if still smoking), number of quit attempts since baseline, and number of uses of other smoking-cessation aids since baseline.

#### Mediator Outcomes

Mediator outcomes include the confidence in being able to quit smoking (self-efficacy; measured on a scale from 1 to 10), importance of quitting (scale from 1 to 10), and knowledge of how to quit smoking (scale from 1 to 10).

### Participant Timeline

**Figure 1:**
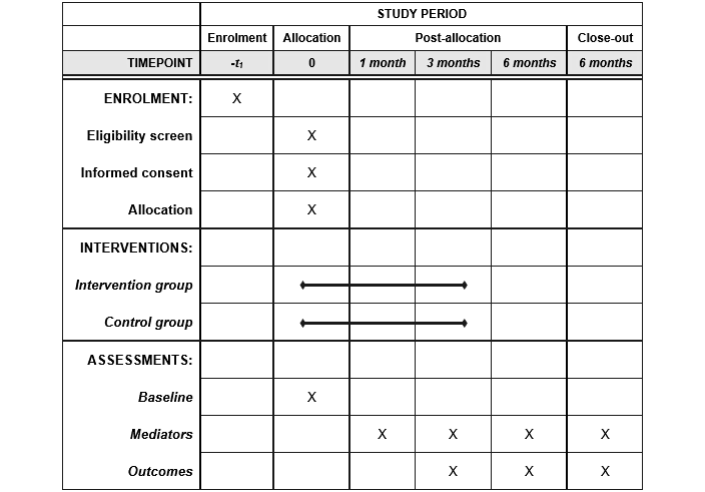
SPIRIT figure representing participant progress throughout the trial.

A timeline for participants’ progress throughout the trial is presented in Figure 1. The baseline questionnaire will assess for eligibility, and after completion, participants will be immediately randomized. The intervention period will last for 3 months, and mediator and outcome measures will be assessed at 3 months and 6 months after randomization. Mediator outcomes will also be assessed 1 month after randomization. Participation is complete after the 6-month assessment.

### Assignment of Interventions

Allocation will be done according to a computer-generated random sequence. Prior to randomization, participants will be stratified according to which of the 2 versions of the intervention is appropriate (general or surgery). Block randomization will be used to ensure equal number of participants in each group within stratum. Random block sizes of 2 and 4 will be used in order to prevent subversion of allocation concealment.

Randomization will be done immediately after responding to the baseline questionnaire, which is done by participants on their mobile phones. Once responses are received by the backend server, automatic randomization will take place, and participants will be told about group allocation via a text message. Research personnel will not be able to affect the allocation.

Participants will be aware of their group allocation; however, research personnel will be blinded. All questionnaires are completed by participants on their own mobile phones, without supervision by research personnel. These automated procedures ensure no unblinding. Nonresponders to questionnaires will be called by phone (see Data Collection), and during this time, it is possible that participants will reveal their allocation to assessors (see Generalization and Limitations).

### Data Collection

Baseline questionnaires will be completed by participants on their mobile phones at the time of enrollment. There are 3 follow-up intervals: 1 month, 3 months, and 6 months after randomization. All follow-ups will be initiated by sending text messages to participants with hyperlinks to questionnaires. Only mediators will be assessed at the 1-month follow-up; there will be no smoking cessation outcomes. In all cases, the following attempts will be made to collect data:

1. A total of 2 reminders will be sent 2 days apart to those who have not responded.
2. If no response is given to (1), then we will send questions directly in a text message, asking participants to respond directly with a text (no hyperlink). At 1 month, we will ask all 3 mediator questions. At 3 months and 6 months, we will only ask for primary outcome measures.
3. If there is no response given to (2) at 3 months and 6 months, we will attempt to call participants to collect responses to the same questions as in (2). No phone calls will be made to collect 1-month follow-up data. A maximum of 5 call attempts will be made.

The 2 smoking cessation outcome follow-up intervals of 3 months and 6 months measure the immediate effect of the intervention and the prolonged effect of the intervention, respectively. As such, we are not proposing either to be primary above the other. Note that since our analyses are not primarily based on null hypothesis testing (see Statistical Analyses), we are not majorly concerned about the increased error rate by having multiple primary follow-up intervals.

### Statistical Analysis

#### Overview

All randomized individuals will be included in analyses, following intention-to-treat principles. Missing data will initially be handled by available case analysis under the missing completely at random (MCAR) assumption. Systematically missing data will invalidate the MCAR assumption; thus, evidence of such will be sought. If data are missing systematically, then it may be the case that early responders differ from nonresponders and, in extension, that late responders are more like nonresponders. Therefore, one analysis will regress primary outcomes against the number of attempts to collect follow-up data before a response was recorded. Attrition analyses will further explore the MCAR assumption by investigating if responders and nonresponders are different with respect to baseline characteristics.

We anticipate approximately a 10%-20% attrition rate, as this is what we have experienced in previous trials of text messaging interventions in Sweden when we used a similar scheme for data collection [11,14,26-28]. We have no reason to believe that attrition rates will be different between recruitment settings, as all follow-up procedures will be the same, but we will investigate such differences and report our findings in light of them. Sensitivity analyses that include imputed values for missing outcome data will be performed, and limitations of the imputed analyses will be considered in face of the actual attrition rate. In addition, data will be graphically examined for outliers or data input errors, and sensitivity analyses will be performed excluding any erroneous data points.

We will estimate all models using Bayesian inference [29-31] and report the marginal posterior probability of an effect of group allocation on each of the outcomes. We will use the median as a point estimate of the effect and report 95% compatibility intervals. We will complement the Bayesian inference with null hypothesis tests at the .05 significance level. Both posterior distributions and significance tests will create a basis for scientific inference.

#### Models

Baseline characteristics will be compared between the intervention and control groups using Fisher exact tests and Mann-Whitney U tests. Using logistic regression, we will compare characteristics of participants recruited through the online setting versus those through facilitated recruitment and between those eligible for the general version versus the surgery version of the intervention.

For the primary and secondary outcome measures, differences between the 2 groups (control and intervention) at the different follow-up stages with respect to prolonged abstinence, point prevalence of smoking abstinence, and 7-day point prevalence will be analyzed using logistic regression. Negative binomial regression will be used to analyze the number of quit attempts, use of other smoking cessation services, and cigarettes smoked weekly (among those who still smoke). Models will be adjusted for baseline characteristics (gender, age, nicotine dependence, importance, self-efficacy, and know-how) as well as the stratifying variable in the randomization procedure (general or surgery eligibility).

Effect-modification analyses will be performed for the 2 primary outcomes. The following potential effect modifiers measured at baseline will be explored: gender, age, years of smoking, mean number of cigarettes smoked weekly, use of snus, nicotine dependence, importance, self-efficacy, and know-how. In addition, effect modification based on which setting (online or primary health care) participants were recruited will be explored and which version of the intervention they were eligible for (general vs surgery). Effect-modification analyses will be performed by including interaction terms in the adjusted regression models for each potential moderator (one model per moderator).

For the mediator outcomes, mediators will be explored using a causal inference framework [32-34] using Bayesian inference to estimate the natural direct effect and natural indirect effect (as per the definitions of Pearl [34]). We will report on the posterior distributions of these 2 estimates, as well as the proportion of the total effect that is accounted for by the natural indirect effect. Four models will be created for each primary outcome measure, 3 investigating the mediating factors on their own and a fourth incorporating all mediators at once. If any baseline characteristics are found to moderate the effect in the primary analysis, then additional mediator models will be created to include these as moderators.

#### Exploratory Analyses

RCTs traditionally contrast 2 or more groups, however do not address individual variability (also known as heterogenous treatment effects). Some individuals may respond well to an intervention, while others might not, and some may be harmed; however, contrasting 2 heterogenous groups does not identify such differences. Predicting how individuals will respond to an intervention using baseline characteristics is one way of early identification of individuals who may benefit, but also allows us to identify groups of individuals who are less likely to respond well to the intervention [35]. We will therefore learn prediction models from the trial data to predict outcome given baseline values and use a combination of clustering and multinomial regression to identify and label groups of individuals who may be more or less helped by the novel intervention.

We will investigate differences in outcomes between control subjects with respect to the 2 modes of recruitment (online and primary health care). All control subjects will receive links to online resources; however, those who have been recruited at primary health care units will in addition be told about resources available at the unit. Also, participants may react differently to being allocated to the control arm (see Generalizability and Limitations). Thus, these analyses will help to inform potential biases in effect estimates and to inform if offering additional support at the unit is effective above online resources.

### Sample Size

We will use a Bayesian group sequential design to monitor recruitment with interim analyses planned every other week after the first 20 participants have completed the 6-month follow-up. Each of the primary outcomes (prolonged abstinence and point prevalence) will be modelled according to the analysis plan (see Statistical Analysis), and the coefficient for group allocation will be assessed for effect, harm, and futility. Let ß_k,i_ represent the regression coefficient for group allocation at time k for outcome i and D all the data currently accumulated, then the target criteria will be: p(ß_k,i_ > 0 | D) > 97.5% and p(ß_k,i_ > log(1.3) | D) > 50% for effect; p(ß_k,i_ < 0 | D) > 97.5% and p(ß_k,i_ < log(1/1.3) | D) > 50% for harm; and p(log(1/1.3) < ß_k,i_ < log(1.3) | D) > 95% for futility.

For the effect and harm criteria, we will use a standard normal prior for dummy covariates (mean 0, SD 1.0) and a slightly wider prior will be used for the futility criterion (mean 0, SD 2.0). The criteria should be viewed as targets; thus, at each interim analysis, we will evaluate each criterion for each covariate and make a decision if we believe that recruitment should stop or continue. However, recruitment will not exceed 24 months.

Note that this Bayesian approach allows us to look at the data an unlimited number of times without worrying about multiplicities and error rates, as would be necessary using a frequentist approach [36]. Also, since no fixed effect size is prespecified, we reduce the risk of stopping both too early and too late [21].

## Results

Recruitment commenced in September 2020 and will not exceed 24 months. This means that a complete dataset will be available at the latest towards the end of 2022. We expect to publish the findings from this trial by June 2023.

## Discussion

### Effects of text messaging interventions

This trial will further our understanding of the effects of text messaging interventions among a more general population than has previously been studied. We also aim to learn about differential effects between those who seek support online and those who are given facilitated support at primary health care units.

We expect to expand upon current knowledge on how text messaging interventions may work by the investigation of mediators. The text2quit trial found that self-efficacy, know-how, and the sense that somebody cared partially mediated the intervention’s effect on smoking cessation [37]; however, there are no other similar mediator studies. Thus, the body of evidence needs to be expanded. Finally, we will also investigate for whom the interventions work by analyzing heterogenous treatment effects.

### Generalizability and Limitations

Trial recruitment is limited to the Swedish population; however, a strength of this study is the pragmatic way in which participants are recruited. Through online advertisements, individuals who decided to look for support without interference by study procedures will be recruited. At primary health care units, individuals who were not necessarily looking for smoking cessation support are given information about the trial. This closely mimics the way the intervention would be disseminated in a real-world setting and may therefore strengthen the argument of generalizability of findings.

The pragmatic design of the study aims to estimate the effects of the public health initiative as a whole (ie, recruiting participants in both online and primary health care settings). These effect estimates are also the target of our Bayesian group sequential design, which dictates the sample size. This design does however limit our ability to estimate differential effects of the 2 versions of the intervention, as well as moderating effects of the study setting. We have however decided upon this pragmatic design as it is uncertain how many participants are in need of the surgery version of the intervention, as well as the recruitment rates in the different settings, thus forming a hypothesis of the differential effect among versions and setting targets for future research.

There are well-known artifacts that arise from knowledge of participation in research that may both dilute and inflate effect estimates [38-41]; thus, these should be considered when generalizing the findings from this and other trials. Participants recruited through online advertisements are actively seeking help, and baseline assessment may therefore be an effective way for them to decide to change their behavior, diluting the effects of the intervention, as there is a ceiling limit for the intervention’s effect. Those recruited through primary health care centers are less likely to be looking for cessation support at the moment, although they may have thought about it in the past, and baseline assessment may make participants allocated to the intervention arm more receptive to the intervention than they would have been otherwise, possibly inflating effect estimates. We will have an opportunity to explore the differences between control and intervention subjects with respect to mode of recruitment to estimate these biases.

The lack of blinding of participants may introduce forms of performance bias, including both movement away and towards behavior change due to disappointment about being in the control group, and while we may hypothesize about the magnitude of this bias, we will consider it a limitation of the trial that we have no means of accounting for quantitatively.

A risk of detection bias stems from the scheme used to decrease attrition bias, by calling participants not responding to initial attempts to collect data (see Data Collection). In such a scenario, it is possible that participants disclose their group allocation to research personnel, who are otherwise blinded, during the follow-up interview. We believe that the advantage of higher follow-up rates gained by calling nonresponders outweighs this risk of bias, and personnel making the calls will be instructed to not prompt and to avoid engaging in conversation about group allocation.

Outcome measures will be self-reported, which may be susceptible to recall and social desirability bias. The Society for Research on Nicotine and Tobacco does however recommend that, in studies with limited face-to-face contact, it is neither required nor desirable to use biochemical verification [25]. Despite this recommendation, results from this trial should be understood under this limitation, as the risk of bias is exacerbated due to participants not being blinded.

### Summary

While the prevalence of smoking in Sweden has decreased over the past decade, people still start smoking, and current smoking cessation aids may not be sufficient to push the prevalence further towards zero. This trial will be the first to estimate the effects of a smoking cessation text messaging intervention among both online help seekers and primary health care patients in Sweden. If effects are found to be important, then dissemination can be quick due to the trial’s pragmatic design, which may help to further reduce the smoking prevalence in Sweden.

This trial also contributes to the overall body of evidence for text messaging interventions, as it looks to increase our understanding of how effects are mediated through psychosocial variables and how effects are differential with respect to passive online recruitment and active facilitated access. Understanding how the effects of text messaging interventions are differential will help us develop more tailored and effective interventions and make support decisions about how to disseminate the interventions into real-world practice.

## Appendix

Multimedia Appendix 1 Informed consent.

Multimedia Appendix 2 Questionnaires.

## Abbreviations

MCAR: missing completely at random
OR: odds ratio
RCT: randomized controlled trial
