# Supplementary material for: Effects of a Text Messaging Smoking Cessation Intervention Among Online Help Seekers and Primary Health Care Visitors in Sweden: Protocol for a Randomized Controlled Trial Using a Bayesian Group Sequential Design
Source: JMIR Res Protoc. 2020 Dec 3;9(12):e23677. doi: 10.2196/23677 (PMC7746491; doi:10.2196/23677)
Supplement: Multimedia Appendix 1 [file resprot_v9i12e23677_app1.docx]

# Informed consent

We are asking you to take part in a research project described below. By pressing the button ”I consent to take part in this study”, you confirm that you have read and agreed to the information given on this page.

**What is the project and why do you want me participate?**The project aims to investigate the effects of a text messaging based smoking cessation intervention among people who want help to quit smoking. The project is led by researchers at Linköping University, who have also been responsible for the development of the intervention. The content of the intervention is based on both previous research and practical experience from smoking cessation programs in Sweden.

**What does participation entail?**If you decide to participate, then you will first be asked to respond to a brief questionnaire, and then you will be randomly allocated to one of two groups. Only one of the groups will be given access to the text message intervention. Regardless of which group you have been allocated to, you are free to use all other types of smoking cessation support that you have access to.

The intervention consists of a series of text messages that will be sent to your mobile phone. The messages sent the first week will prepare you to quit. After the first week you will be given tips on how to deal with initial cravings, and exercises to help you deal with withdrawal symptoms. The number of messages will reduce week by week, and the program lasts for a total of 12 weeks. You can always text the word STOP to us if you want to stop receiving text messages.

Regardless of which group you have been allocated to, all participants will be asked to respond to a brief questionnaire at 1, 3 and 6 months after initially signing up for the study. This is necessary for us to be able to estimate the effects of the intervention.

**Consequences from taking part in the study**We do not believe that there are any risks for you as an individual in taking part in this study. Participation does not imply that you have to refrain from using any other support available to you. You can at any time decide to leave the study. Questions about the study can be directed to the primary investigator (see contact details at the end of this page).

**What will happen to my personal information?**Throughout the project we will collect data from you. The objective with this data collection is research, and is legally justified due to public interest. Data from questionnaires and interactions with the intervention will be stored. We will keep your data connected to an encrypted version of your phone number, which requires a secret key to decrypt. Only research personnel will have access to this secret key, and all data will be stored on a database at Linköping University. We will only report results at the group level. When the project is finalized we will delete your phone number, which means that it will no longer be possible to trace data to individuals.

Your data will be treated with confidentiality, and Linköping University will be responsible for all personal data. In accordance with GDPR (EU 2016/679), you can at any time be given access to your personal data that we store (free of charge). You can also ask for corrections to be made, or for your data to be deleted entirely. Please contact the primary investigator if you want to access to your data. You can also contact the data protection officer at dataskyddsombudet@liu.se. If you are not satisfied with the way your personal data has been handled, you can file a complaint at the Swedish Data Protection Authority.

Please note that once the project has been finalized only anonymized data will be stored. This means that we will no longer be able to trace your individual data. Data will be retrieved and deleted from the database and stored on secure file storage at Linköping University.

Text messages that are sent as part of the study will be handled by a private company called Alexit AB. The company is owned by the primary investigator of this study. Alexit AB will only help with the sending of text messages and not have access to any of the data collected in the research project.

**How can I get information about results the study?**When the study is finalized we will publish the results in scientific peer-reviewed open access journals. Results will never be traceable to individuals from these publications. You can always contact the primary investigator if you want to know more about the results of the study.

**Insurance and remuneration**No remuneration will be given to participants. Insurance is available through Kammarkolegiet by Linköping University.

**Participation is free and non-mandatory**Your participation is free and non-mandatory, and you can at any time decide to leave the study. If you decide that you no longer want to take part, then you do not have to tell us why, and it will not affect or restrict any of the healthcare services provided to you.

**Research group**Professor Preben Bendtsen, preben.bendtsen@liu.se (primary investigator)
Dr Marcus Bendtsen, marcus.bendtsen@liu.se
Dr Kristin Thomas, kristin.thomas@liu.se
Linköpings Universitet, Institutionen för Hälsa, Medicin och Vård
