# Supplementary material for: Effects of a Text Messaging Smoking Cessation Intervention Among Online Help Seekers and Primary Health Care Visitors in Sweden: Protocol for a Randomized Controlled Trial Using a Bayesian Group Sequential Design
Source: JMIR Res Protoc. 2020 Dec 3;9(12):e23677. doi: 10.2196/23677 (PMC7746491; doi:10.2196/23677)
Supplement: Multimedia Appendix 2 [file resprot_v9i12e23677_app2.docx]

## Baseline questionnaire

1. Gender:
   1. Female
   2. Male
   3. Other
2. Age (numerical measure)
3. Do you have surgery planned within the next 3 months?
   1. Yes (Go to Question 4)
   2. No or unsure (Go to Question 6)
4. Do you know the date of your surgery?
   1. Yes (Go to Question 5)
   2. No (Go to Question 6)
   3. Unsure (Go to Question 6)
5. In how many weeks will you undergo surgery? (numerical measure)
6. How many years have you smoked? (numerical measure)
7. How many cigarettes do you smoke per day/week/month? (numerical measure)
8. Do you use snus?
   1. No
   2. A few times each month
   3. A few times each week
   4. Daily – less than 1/3 box
   5. Daily – 1/3 box
   6. Daily – 1/2 box
   7. Daily – 1 box or more
9. How long after you have woken up do you smoke your first cigarette?
   1. Within 5 minutes
   2. After 6-30 minutes
   3. After 31-60 minutes
   4. After more than 60 minutes
   5. I don’t smoke
10. Do you struggle to stay smoke free in situations where it is not allowed to smoke, e.g. at the cinema or during flights?
    1. Yes
    2. No
11. Which cigarette is hardest to stay away from?
    1. The one in the morning
    2. Any other cigarette
12. Do you smoke more during the mornings than during the rest of the day?
    1. Yes
    2. No
13. Do you smoke even if you are so ill that you are bedridden?
    1. Yes
    2. No
14. How many times have you tried to quit smoking? (numerical measure)
15. Have you ever received professional help, individually or in a group, to quit smoking?
    1. Yes, I am receiving help at the moment
    2. Yes, but not right now
    3. No
16. Have you ever called the quit smoking hotline (Sluta-Röka-Linjen)?
    1. Yes
    2. No
17. How confident are you that you will be able to quit smoking? (10-point scale ranging from 1 = “Not at all” to 10 = “Very confident”)
18. How important is it for you to quit smoking? (10-point scale ranging from 1 = “Not important” to 10 = “Very important”)
19. How well do you know how to quit smoking? (10-point scale ranging from 1 = “Not well at all” to 10 = “Very well”)

## Mediation questionnaire (1 month)

1. How confident are you that you will be able to quit smoking? (10-point scale ranging from 1 = “Not at all” to 10 = “Very confident”)
2. How important is it for you to quit smoking? (10-point scale ranging from 1 = “Not important” to 10 = “Very important”)
3. How well do you know how to quit smoking? (10-point scale ranging from 1 = “Not well at all” to 10 = “Very well”)

## Follow-up questionnaire (3- and 6- months)

1. (Asked to surgery participants only) Have you had your surgery?
   1. Yes (Go to Question 2)
   2. No, but I know approximately when I will have it (Go to Question 3)
   3. No, and I am not sure that the surgery will happen (Go to Question 4)
2. (Asked to surgery participants only) How many weeks ago did you undergo surgery? (numerical measure)
3. (Asked to surgery participants only) In how many weeks do you think you will undergo surgery? (numerical measure)
4. Have you smoked more than 5 cigarettes the past 8 weeks? (8 weeks will change to 5 months at the 6- month follow-up).
   1. Yes
   2. No
5. Have you smoked any cigarette the past 4 weeks?
   1. Yes
   2. No
6. Have you smoked any cigarette the past 7 days? (Only asked if a) to Question 5)
   1. Yes
   2. No
7. How many cigarettes do you smoke per day/week/month? (numerical measure) (Only asked if a) to Question 6)
8. Do you use snus?
   1. No
   2. A few times each month
   3. A few times each week
   4. Daily – less than 1/3 box
   5. Daily – 1/3 box
   6. Daily – 1/2 box
   7. Daily – 1 box or more
9. How many quit attempts have you made since you joined the trial? (numerical measure)
10. Have you sought any other support to quit smoking since you joined the trial? (Multiple answers can be given) (Several options with available support mechanism, including hotline, prescription drugs, etc.)
11. How confident are you that you will be able to quit and stay smoke free? (10-point scale ranging from 1 = “Not at all” to 10 = “Very confident”)
12. How important is it for you to quit and stay smoke free? (10-point scale ranging from 1 = “Not important” to 10 = “Very important”)
13. How well do you know how to quit and stay smoke free? (10-point scale ranging from 1 = “Not well at all” to 10 = “Very well”)
